# Antithrombin III (AT) and recombinant tissue plasminogen activator (R-TPA) used singly and in combination versus supportive care for treatment of endotoxin-induced disseminated intravascular coagulation (DIC) in the neonatal pig

**DOI:** 10.1186/1477-9560-4-7

**Published:** 2006-05-18

**Authors:** Rachel Davis-Jackson, Hernan Correa, Ronald Horswell, Halina Sadowska-Krowicka, Kathleen McDonough, Chittaranjan Debata, Renee' Gardner, Duna Penn

**Affiliations:** 1 Louisiana State University Health Sciences Center – Earl K. Long Medical Center, 5825 Airline Hwy, LSU Unit Baton Rouge, Louisiana (La.), 70805, USA; 2 Louisiana State University Health Sciences Center – New Orleans Children's Hospital Dept. of Pathology, 200 Henry Clay, New Orleans, La, 70118, USA; 3 Louisiana State University Health Sciences Center – Pennington Biomedical Research Center, 6400 Perkins Rd, Baton Rouge, La 70808, USA; 4 Louisiana State University Health Sciences Center – New Orleans, Dept. of Pediatrics, Research Institute for Children, 200 Henry Clay Ave, New Orleans, La 70118, USA; 5 Louisiana State University Health Sciences Center – New Orleans, Dept. of Physiology, 1901 Perdido Street, New Orleans, La 70112, USA; 6 Louisiana State University Health Sciences Center – New Orleans, Dept. of Pediatrics, Children's Hospital, 200 Henry Clay Ave, New Orleans, La 70118, USA

## Abstract

**Background:**

Disseminated intravascular coagulation (DIC) is a pathological disturbance of the complex balance between coagulation and anticoagulation that is precipitated by vascular injury, acidosis, endotoxin release and/or sepsis and characterized by severe bleeding and excessive clotting. The innately low levels of coagulation factors found in newborn infants place them at extremely high risk for DIC. Anecdotal reports suggest that either anticoagulant or fibrinolytic therapy may alleviate some of the manifestations of DIC. To test the hypothesis that replacement of both anticoagulants and fibrinolytics may improve survival and outcome better than either single agent or supportive care alone, we utilized a neonatal piglet model of endotoxin-induced DIC.

**Methods:**

DIC was induced in twenty-seven neonatal pigs (7 to 14 days of age) by intravenous administration of E. coli endotoxin (800 μg/kg over 30 min). The piglets were divided into 4 groups on the basis of treatment protocol [A: supportive care alone; B: Antithrombin III (AT, 50 μg/kg bolus, 25 μg/kg per hr continuous infusion) and supportive care; C: Recombinant Tissue Plasminogen Activator (R-TPA, 25 μg/kg per hr continuous infusion) and supportive care; D: AT, R-TPA and supportive care] and monitored for 3 primary outcome parameters (survival time, macroscopic and microscopic organ involvement) and 4 secondary outcome parameters (hematocrit; platelet count; fibrinogen level; and antithrombin III level).

**Results:**

Compared with supportive care alone, combination therapy with AT and R-TPA resulted in a significant improvement of survival time, hematocrit, AT level, macroscopic and microscopic organ involvement, p < 0.05. Compared with supportive care alone, R-TPA alone significantly reduced macroscopic organ involvement and AT alone increased AT levels.

**Conclusion:**

The findings suggest that combining AT, R-TPA and supportive care may prove more advantageous in treating the clinical manifestations of DIC in this neonatal pig model than either single modality or supportive care alone.

## Background

DIC is a combination of enhanced thrombosis and decreased fibrinolysis. It is precipitated by some underlying disorder, e.g. sepsis, that results in production and release of proinflammatory cytokines. These activate the coagulation cascade, inhibit physiologic anticoagulant pathways, and depress fibrinolysis, resulting in enhanced fibrin formation and impaired fibrin removal [1].

Initiated by either contact phase or tissue factor activation, the coagulation cascade is a set of reactions involving multiple coagulation factors and cofactors [2] that result in the generation of thrombin which acts on the fibrin polymer to produce a fibrin clot [3]. This process is subject to regulation by intrinsic inhibitors, e.g. AT and protein C. One of the major inhibitors of coagulation is AT. It rapidly binds and inactivates thrombin and factor Xa by forming thrombin-antithrombin and factor Xa-antithrombin complexes [4]. Protein C is a vitamin K-dependent inactive enzyme precursor that when activated, proteolytically inactivates the activated factors VIII and V by way of cleavage reactions.[5]

The fibrinolytic pathway is a set of reactions involving fibrinolytic factors and cofactors [2] that clear unneeded fibrin from intravascular and extravascular sites [4]. During fibrinolysis, plasminogen is cleaved by tissue plasminogen activator (TPA) or urokinase-type plasminogen activator to form plasmin that lyses the fibrin clot, releasing fibrin degradation products. During DIC, thrombin generation proceeds via the extrinsic tissue factor/factor VIIa route with simultaneous depression of inhibitory mechanisms, such as AT and Protein C [1] and enhanced inhibition of fibrinolysis by plasminogen activator inhibitor (PAI-1) [6].

Neonates are innately predisposed to acquire DIC due to altered levels of coagulation and fibrinolytic proteins that differ markedly from those in adults [5,7]. In neonates, AT levels are within the concentration range at which spontaneous thromboses could occur in adults. Protein C and Protein S are reduced at birth to 30% of adult plasma concentrations and remain decreased during the first weeks of life [5]. In addition, differences in plasma concentrations of fibrinolytic proteins may also contribute to predisposition for DIC [7]. At birth, newborn plasminogen levels are 58% of adult concentrations while plasma concentrations of PAI-1 and TPA are approximately twice adult concentrations and decrease to adult plasma concentration values by day 5 of life [5]. Impaired fibrin degradation, due to high circulating levels of PAI-1, contributes to enhanced intravascular fibrin deposition [1].

Furthermore, sepsis, a major risk factor for DIC, occurs in infants with an incidence of 1 to 5 per 1,000 live births. Mortality is high in the septic neonate, up to 50% for early onset-sepsis [8]., and survivors often exhibit increased morbidity. Hypercoagulability is often seen during sepsis. Fibrin deposition leads to microvascular and less frequently, large vessel thrombosis with impaired perfusion and subsequent organ damage [9]. These microvascular thrombi in various organs contribute to the development of multiorgan failure [4]. Neonates surviving an episode of DIC may be left with the burden of such sequelae and diminished quality of life. For these reason, effective therapy for DIC would have tremendous impact.

Unfortunately, the therapy for DIC in the neonate is by no means optimal. The current mainstay of therapy is "treatment of the underlying illness" and supportive care. The efficacy of coagulant replacement therapy in the overall treatment of DIC in the septic infant is controversial. Gross, et al. reviewed a group of infants with sepsis and DIC and concluded that successful outcome depended on treatment of the underlying illness and that therapy directed toward the coagulopathy did not result in improved outcomes [10]. However, Gross used a nonspecific global approach by administering fresh frozen plasma to replace diminished coagulation factors.

Specific treatment directed toward either the coagulation or fibrinolytic derangement has occasionally been attempted. Previous studies suggest that a unilateral approach to the coagulation or fibrinolytic deficiencies may be of some benefit (Table 1). Most of the human studies were performed in children or adolescents. Because of ethical concerns, controlled studies with neonates are difficult and limited data are available. The animal studies performed so far have focused mainly on correction of anticoagulation defects, although Munoz, et al. addressed both the anticoagulation and fibrinolytic abnormality using a rabbit model [11]. The current study utilizes a newborn piglet model of endotoxin-induced DIC to mimic sepsis and examines the effect of replacing AT and R-TPA singly and in combination. Both are currently available for human use. The newborn piglet was chosen as it is the closest non-primate animal model of the human neonate with respect to anatomy, physiology, biochemistry and metabolism [12]. The hemostatic system in the pig is very similar to that of the human adult[13,14]. Table 2 compares normal coagulation values with reference intervals for the pig and human [15]. Massicotte, et al. considered the newborn piglet to be a suitable model for investigation of specific hemorrhagic and thrombotic problems in the newborn [13].

**Table 1:** Summary of selected therapeutic approaches using replacement of specific anticoagulation and fibrinolytic proteins to treat DIC. This table is based on a PubMed search using the key words: "Disseminated Intravascular Coagulation", "Therapy for DIC", "Animal experimentation on DIC" and "Neonate or Newborn." A representative selection was chosen from a total of 98 papers in the period 1970 to 2005 to give an overview of the literature on this subject.

**Subject**	**Mortality Rate**	**N**	**Therapeutic Factor**	**Provoking Factor**	**Outcome**	**Reference**
**Coagulation Pathway**						
Children	not addressed	4	Protein C	meningococcemia	reversed organ dysfunction	[16]
Infants, adolescents	25%	8	Protein C	meningococcemia	restored micro-circulation	[17]
Children 1 mo-5 yr	17%	6	AT	sepsis, carcinoma, Reye's	normalized coagulation studies	[18]
Newborns	40%	10	AT, heparin	sepsis	improved coagulation	[22]
Child	100%	1	AT, Protein C	Unknown, ARDS	stopped bleeding	[19]
Baboons	67–100%	3	Protein C	*E. coli *organisms	prevented coagulopathy, lethal effects	[25]
Adult pigs	not addressed	16	AT	*E. coli *endotoxin	Improved MAP, SVR	[23]
Guinea pigs	0% with Tx	58	Antithrombin Concentrate & LMWH	*S. aureus *isolates	Decreased fibrin deposits, improved survival	[26]
Adult pigs	Not addressed		AT	LPS	decreased DIC, improved survival	[21]
Adult pigs	Not addressed	21	AT	LPS	reduced effusion, edema	[27]
Newborn and 3 wk old piglets	Not Addressed	>>16/age group & dose	Heparin	^125^I-Fibrinogen	↑ AT conc. Improved the antithrombotic effects of heparin	[28]
**Fibrinolytic Pathway**						
Infant, 4 mo	0%	N 1	R-TPA	meningococcemia	improved organ perfusion, cardiac performance	[24]
Infants	0%	2	R-TPA	Meningococcemia	Improved micro-circulation	[20]
**Combined Coagulation and Fibrinolytic Pathways**						
Adult rabbits	70 → 40% R-TPA alone, 70 → 0% both factors	N	R-TPA, R-hirudin	endotoxin	Improved hemostatic markers, decreased fibrin deposits	[11]

**Legend: **ARDS Adult respiratory distress syndrome

E. Coli Escherichia Coli

kg kilogram

LMWH Low molecular weight heparin

MAP Mean Arterial Pressure

SVR Systemic Vascular Resistance

S. aureus Staphylococcal aureus

Tx Therapy

R-hirudin Recombinant Hirudin

N Subject number

**Table 2:** Porcine and Human Coagulation Values and Reference Ranges [7,15,5]

	**Porcine Juvenile**	**Human Premature Neonate**	**Human Term Neonate**	**Human Adult**
Parameter	Mean ± SD	₤ Mean ± SD Range (mean)	₤ Mean ± SD Range (mean)	Mean ± SD

Hct %	28.89 ± 3.88	₤ 60 ± 8	₤ 61 ± 7	41 ± 3.0
Plt G/L	407.32 ± 121.24	10.6–16.2 (13.0)	10.1–15.9 (13.0)	265.0 ± 57.5
AT %	100.89 ± 11.43	0.14–0.62 (0.38)	0.39–0.87 (0.63)	100.0 ± 10.0
Fib mg/100 ml	357.44 ± 88.43	1.5–3.73 (2.43)	1.67–3.99 (2.83)	285.0 ± 47.5

The major goal of this experiment was to test the hypothesis that replacement of both AT and R-TPA would improve survival and decrease the severity of the disease in the neonate better than either single agent or supportive care alone.

## Methods

Twenty-seven newborn piglets, 7–14 days old, were randomly divided into four groups on the day of experiment (Table 3). Each group received supportive care with or without some other form of therapy. Supportive care included maintenance intravenous fluids (5% dextrose and 1/2 normal saline, 150 ml/kg/day); respiratory support (tidal volume 15 ml/kg, ventilator rate 20/min, FiO2 1.0); volume expansion for decreased mean blood pressure (BP < 30 mm Hg), or central venous pressure (CVP < 5 cm H_2_O) and/or urine output (< 1 ml/kg/hr for more than 2 hours); and dopamine/dobutamine administration (5–15 μg/kg per min) for hypotension unresponsive to a cumulative volume expansion of 60 ml/kg. Group A received supportive care only. Group B received AT (50 μg/kg bolus, and 25 μg/kg continuous intravenous infusion) and supportive care. Group C received R-TPA (0.025 mg/kg/hr) and supportive care. Group D was the combination therapy group receiving AT, R-TPA and supportive care.

**Table 3:** Description of study groups.

**Group**	**(n)**	**AT**	**R-TPA**	**Supportive Care**
**A (control)**	9	No	No	Yes
**B**	6	Yes	No	Yes
**C**	6	No	Yes	Yes
**D**	6	Yes	Yes	Yes

**Legend: **AT: antithrombin III (50 μg/kg bolus, and 25 μg/kg continuous intravenous infusion); R-TPA: recombinant tissue plasminogen activator (0.025 mg/kg/hr).

After anesthesia was induced with intravenous pentobarbital, each piglet was surgically equipped with an endotracheal tube for assisted ventilation, as well as central venous, arterial and bladder catheters. After an initial stabilization period of one hour, DIC was induced by injecting *Escherichia Coli *endotoxin (800 μg/kg over 30 min). Two animals in Group A received 1000 μg/kg. As there were no differences in response noted between them and the 7 animals that received 800 μg/kg of endotoxin, they were included in the analysis. Vital signs and urinary output were continuously monitored. Blood pressure, amount of fluid resuscitation and pressor use were measured to assess our success in maintaining mean BP greater than 30 mm Hg, as per protocol. Serial hematologic and coagulation studies were performed (at 30 min, 1, 2, 4, 6 and 7 hours). After 7 hours, surviving animals were euthanized by exsanguination under deep anesthesia.

The dosages of endotoxin, AT and R-TPA were selected on the basis of review of the literature and pilot studies. Based on studies by Dickenite et al. (24) and Taylor et al. (21) initial doses of endotoxin ranging from 66 to 600 μg/kg were tested. Since these dosages did not produce hemotologic evidence of DIC, the dosage was increased to 800 micrograms/kg which gave definite and reproducible hematologic evidence of DIC and manageable hypotension in most cases.

The dosage for R-TPA was based on studies by Munoz, et al. (15). As doses of R-TPA of 0.2 mg/kg/hr and 0.1 mg/kg/hr were associated with ascites and hemorrhage in various organs, the dosage was decreased to 0.025 mg/kg/hr.

Finally, the AT dosage was derived from Dickenite et al.(23) and tested in pilot studies of AT dosages ranging from 1 unit/kg/hr to 73 units/kg/hr with and without and boluses ranging from 5 to 250 units/kg. The lower dosages had no effects upon clinical condition and AT levels. The highest doses improved AT levels, but were associated with increased hemorrhage. Eventually the dose of 50 units/kg bolus and 25 units/kg/hr continuous drip was chosen to maximize beneficial effect and minimize side effects.

At necropsy, macroscopic organ involvement was subjectively rated by the researchers for visible evidence of hemorrhage in the right or left kidney, heart and liver. Scores of 0–4 were given denoting the number of organs with visible evidence of macroscopic hemorrhage. Tissue samples were collected for histological examination.

Piglet kidney sections were stained with hematoxylin and eosin and evaluated microscopically in a blinded fashion by a single pathologist, Dr. Hernan Correa. All samples were assigned values on a scale from 0–5 (0: no involvement; 1: minimal; 2: mild; 3: moderate; 4: moderately severe; and 5: severe) for two different categories: congestion which was defined by presence and amount of fluid accumulation; hemorrhage which was defined by evidence and degree of bleeding. In addition, thrombosis was rated by presence (+1) or absence (0) of clot formation.

Primary outcome parameters included survival time, macroscopic and microscopic organ involvement and secondary outcome parameters included hematocrit; platelet count; fibrinogen level; and AT level. Blood pressure was a clinical variable measured to assess our success in maintaining mean BP greater than 30 mm Hg, as per protocol. Each treatment factor (AT, R-TPA), was tested at two levels. The first level compared the factor alone against supportive care alone and the second level compared the combined factors against supportive care alone. Statistical analysis of the data was undertaken using matched pair t-tests to compare baseline to follow-up changes for platelets, fibrinogen, AT, blood pressure and hematocrit level within each of the four groups. A permutation procedure was used to calculate significance levels due to non-normality and heteroskedasticity across treatment groups for blood pressure. A permutation test was also used to compare groups on number of organs displaying macroscopic organ involvement. Survival time across groups was compared using a survival model assuming exponentially distributed survival times with survival time censored at seven hours. *P*-values are from one-way statistical tests and are not adjusted for multiple testing, as these analyses are considered exploratory rather than confirmatory. All analyses were done using Stata 8.2.

This project was reviewed and approved by the Louisiana State University Institutional Animal Care and Use Committee.

## Results

### Clinical parameters

#### Blood pressure

Mean blood pressures fell (*P *< 0.0002) after the administration of endotoxin (Table 4). The average change from baseline to follow-up for BP was statistically analyzed for each group. There were no significant differences among the groups. Table 5 demonstrates the amount of fluids and pressors administered to each group, following parameter protocols for fluid resuscitation and pressor usage. The apparent incongruity noted in Group A requiring the least amount of resuscitation is more than likely secondary to the shortened survival time of these piglets.

**Table 4:** Changes in blood pressure in anesthetized piglets before and after induction of DIC by intravenous administration of endotoxin.

**Mean Blood Pressure ± SD (mm Hg)**				*P*-Value*		
**Group**	**Basal**	**Follow-up**	**Δ**	**B vs.**	**C vs.**	**D vs.**

**A: Supportive care**	74 ± 11	32 ± 23	-42 ± 26	0.238	0.195	0.118
**B: AT**	70 ± 17	25 ± 13	-45 ± 26		0.970	0.382
**C: R-TPA**	70 ± 15	23 ± 13	-47 ± 6			0.375
**D: AT + R-TPA**	65 ± 13	29 ± 4	-36 ± 14			

**Legend**: **Δ **is the average change of follow-up from baseline. AT: antithrombin III (50 μg/kg bolus, and 25 μg/kg continuous intravenous infusion); R-TPA: recombinant tissue plasminogen activator (0.025 mg/kg/hr); SD: standard deviation. *P-values are based on statistical analysis of the average change (Δ) from baseline to follow-up for BP (paired t-test).

**Table 5:** Fluid and pressor usage in the 4 groups of piglets following endotoxin administration.

**Group**	**Fluid Volume (ml)**	**Dopamine**	**Dopamine + Dobutamine**
**A**	107.8 ± 61.7	33%	33%
**B**	82.2 ± 34.6	100%	67%
**C**	83.7 ± 56.8	100%	50%
**D**	121.7 ± 66.8	100%	83%
*P-values	0.559	0.49	0.528

**Legend: **Fluid volume is the mean of total volume given in resuscitative measure for that particular group ± the standard deviation. The use of Dopamine and Dobutamine is expressed as percentage of animals in each group that received pressors. *P-values reflect testing for differences across the groups (Fisher's Exact Test).

#### Survival time

**Figure 1:**
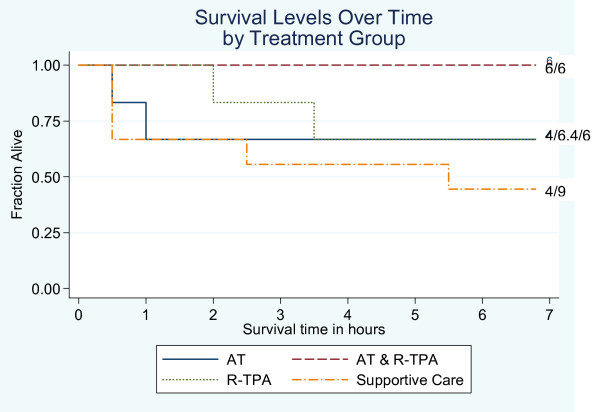
Survival curve by treatment group of anesthetized piglets following DIC induction by intravenous administration of *E. coli *endotoxin (800 μg/kg over 30 min). The numbers on the far right of each survival curve indicate the number of surviving animals after 7 hours as a fraction of the original number. Group A: supportive care only (n = 9); Group B: supportive care + AT (antithrombin III) (n = 6); Group C: supportive care + R-TPA (recombinant tissue plasminogen activator) (n = 6); Group D: supportive care + AT + R-TPA (n = 6).

The survival time findings (Fig. 1) support the research hypotheses showing a tendency for improving survival across the groups (A < B = C < D). Mean survival times (± SD) were 4.16 ± 3.10, 4.92 ± 3.23, 5.58 ± 2.25, and 7.00 ± 0.00 hours for Groups A, B, C, and D, respectively. Mean survival time was only significantly different between Groups A and D (*P *= 0.042), suggesting that the combined effect of AT and R-TPA was greater than either individual effect. However, the small sample size is problematic and limited the power to detect survival time differences statistically. Moreover, censoring survival time at seven hours may have limited detection of variation across the groups. A longer survival time cut-off may have allowed detection of other survival time differences.

### Macroscopic and microscopic organ involvement

**Figure 2:**
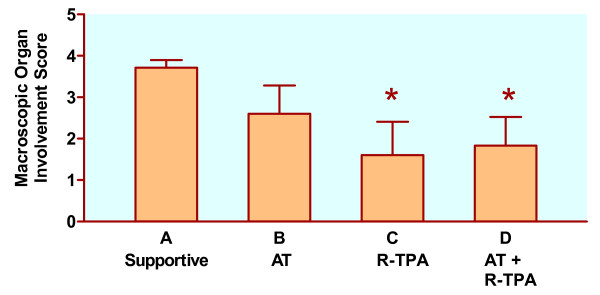
Macroscopic organ involvement after induction of DIC in anesthetized piglets. At necropsy, scores of 0 to 4 were given based on number of organs (right or left kidney, heart and liver) with visible evidence of hemorrhage. AT: antithrombin III (50 μg/kg bolus, and 25 μg/kg continuous intravenous infusion); R-TPA: recombinant tissue plasminogen activator (0.025 mg/kg/hr). Data are expressed as mean ± SD (standard deviation). *P < 0.025, compared with supportive therapy alone.

**Figure 3:**
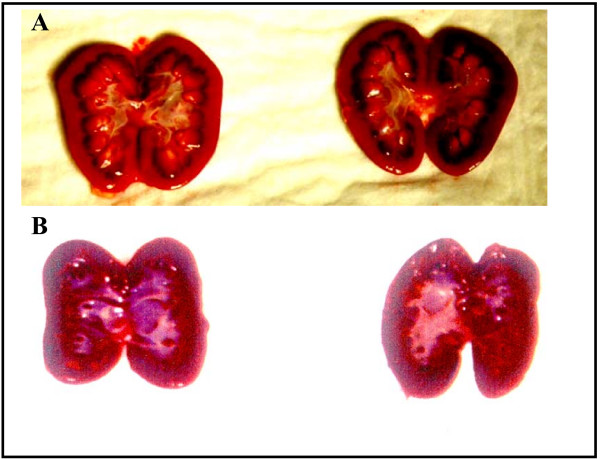
Photograph of right and left kidneys from A) a piglet from Group A that received only supportive care, exhibiting hemorrhages in the renal parenchyma; and B) an animal from Group D that received supportive care, R-TPA and AT. No hemorrhagic changes were seen.

**Figure 4:**
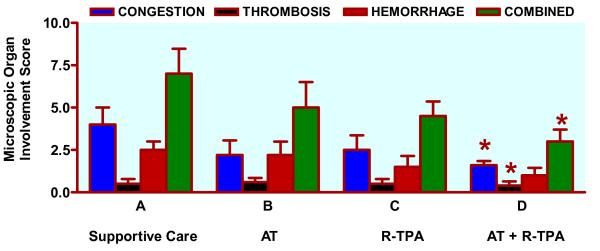
Microscopic organ involvement. After staining with hematoxylin and eosin, piglet kidney histology specimens were evaluated microscopically in a blinded fashion by a single pathologist and assigned values on a scale from 0–5 (0: no involvement; 1: minimal; 2: mild; 3: moderate; 4: moderately severe; and 5: severe) for two different categories: congestion and hemorrhage. Thrombosis was rated by presence (+1) or absence (0). AT: antithrombin III (50 μg/kg bolus, and 25 μg/kg continuous intravenous infusion); R-TPA: recombinant tissue plasminogen activator (0.025 mg/kg/hr). Data are expressed as Mean ± SD (standard deviation). Group A: n = 4; Group B: n = 5; Group C: n = 4; Group D: n = 5. * *P *< 0.05 compared with supportive care alone.

The data (Figure 2) suggest that R-TPA and combined therapy reduce macroscopic organ involvement after induction of DIC. Figure 3 demonstrates typical organ involvement. The data shown in Fig. 4 support our research hypothesis that combined therapy with AT and R-TPA results in less microscopic organ involvement than supportive therapy alone.

#### Hematocrit levels

The average change from baseline to follow-up for hematocrit (Table 6) was statistically analyzed for each group. The pattern of change was consistent with the research hypotheses. Specifically, hematocrit was better preserved in Group D than in Group A (*P *= 0.003) and tended to be better than in Group C (*P *= 0.05).

**Table 6:** Changes in hematocrits of anesthetized piglets before and after induction of DIC by intravenous administration of endotoxin.

**Mean Hematocrit ± SD (%)**				*P*-Value*		
**Group**	**Basal**	**Follow-up**	**Δ**	**B vs.**	**C vs.**	**D vs.**

**A: Supportive care**	26 ± 5	21 ± 8	-5 ± 4	0.155	0.353	**0.003**
**B: AT**	28 ± 9	26 ± 9	-2 ± 7		0.647	0.115
**C: R-TPA**	32 ± 5	28 ± 8	-4 ± 7			0.050
**D: AT + R-TPA**	27 ± 5	29 ± 6	2 ± 4			

**Legend**: **Δ **is the average change of follow-up from baseline. AT: antithrombin III (50 μg/kg bolus, and 25 μg/kg continuous intravenous infusion); R-TPA: recombinant tissue plasminogen activator (0.025 mg/kg/hr); SD: standard deviation. *P-values are based on statistical analysis of the average change from baseline to follow-up for hematocrit (paired t-test).

#### Platelets

The average change from baseline to follow-up in platelet level was analyzed for each group (Table 7). There were no statistically significant differences among the groups although the power was low due to the limited sample size.

**Table 7:** Changes in platelet count of anesthetized piglets before and after induction of DIC by intravenous administration of endotoxin.

**Mean Platelet Count ± SD (× 10^3^/cu. mm)**				*P*-Value*		
**Group**	**Basal**	**Follow-up**	**Δ**	**B vs.**	**C vs.**	**D vs.**

**A: Supportive care**	532 ± 274	179 ± 192	-353 ± 268	0.105	0.392	0.332
**B: AT**	574 ± 133	357 ± 134	-217 ± 79		0.090	0.775
**C: R-TPA**	543 ± 74	207 ± 85	-336 ± 130			0.407
**D: AT + R-TPA**	666 ± 203	408 ± 274	-258 ± 262			

**Legend**: **Δ **is the average change of follow-up from baseline. AT: antithrombin III (50 μg/kg bolus, and 25 μg/kg continuous intravenous infusion); R-TPA: recombinant tissue plasminogen activator (0.025 mg/kg/hr); SD: standard deviation. *P-values are based on statistical analysis of the average change from baseline to follow-up for platelet count (paired t-test).

#### Fibrinogen level

The average change from baseline to follow-up (Table 8) was analyzed for each group. There were no significant differences found.

**Table 8:** Changes in fibrinogen levels of anesthetized piglets before and after induction of DIC by intravenous administration of endotoxin.

**Mean Fibrinogen Level ± SD (mg/dl)**				*P*-Value*		
**Group**	**Basal**	**Follow-up**	**Δ**	**B vs.**	**C vs.**	**D vs.**

**A: Supportive care**	144 ± 23	67 ± 27	-77 ± 34	0.102	0.786	0.571
**B: AT**	156 ± 26	104 ± 32	-52 ± 33		0.055	0.823
**C: R-TPA**	149 ± 31	60 ± 43	-89 ± 26			0.307
**D: AT + R-TPA**	157 ± 36	85 ± 33	-72 ± 52			

**Legend**: **Δ **is the average change of follow-up from baseline. AT: antithrombin III (50 μg/kg bolus, and 25 μg/kg continuous intravenous infusion); R-TPA: recombinant tissue plasminogen activator (0.025 mg/kg/hr); SD: standard deviation. *P-values are based on statistical analysis of the average change from baseline to follow-up for fibrinogen level (paired t-test).

#### AT level

**Figure 5:**
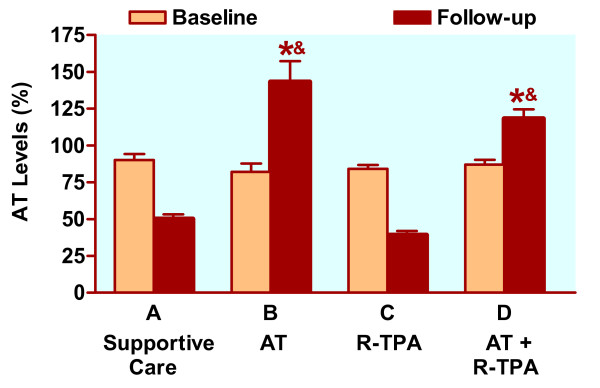
Antithrombin III levels in anesthetized piglets receiving endotoxin. AT: antithrombin III (50 μg/kg bolus, and 25 μg/kg continuous intravenous infusion); R-TPA: recombinant tissue plasminogen activator (0.025 mg/kg/hr). Data are expressed as mean ± SD (standard deviation). * *P *< 0.005 compared with supportive care alone; ^&^*P *< 0.035, compared with Group C (R-TPA alone)

The data (Fig. 5) clearly demonstrate a positive effect of AT administration upon post-endotoxin AT levels and support the research hypothesis.

## Discussion

DIC is a life-threatening disease with serious mortality and morbidity. Controversy exists concerning the efficacy of various treatment modalities using coagulation factor replacement [16].

Most previously published studies [17-20] used a monotherapeutic approach directed at either the anticoagulation or fibrinolytic derangement and were performed in children, adolescents or adults. There have been very few reports in the literature concerning such therapies in the neonate [20,22]. Yet, neonates, and especially premature infants, are at increased risk for DIC due to developmental differences in their levels of coagulation and anticoagulation factors at birth [4]. This study explored the efficacy of a dual anticoagulation and fibrinolytic agent replacement therapy in the newborn piglet model of DIC.

Although hampered by a small sample size that limited the power to demonstrate statistical significance, our study results from the first level of analysis, i.e. factor alone compared with supportive care, showed a trend toward improved survival and less severe hematologic and coagulation system derangements that are comparable with past studies. Dickneite, et al. [21] demonstrated that AT administered to adult pigs in porcine septic shock decreased DIC and improved survival. Von Kries, et al. [22] reported that AT administration improved the coagulation derangement in 10 newborns with sepsis. In contrast to Fourier, et al [23] who reported improved mean arterial pressure and systemic vascular resistance when AT was used after *E. Coli *endotoxin was administered to adult pigs, we found no beneficial effect of AT on blood pressure in our piglets. This may reflect differences in cardiovascular response between neonatal and adult responses in the pig, but could also just be due to the small numbers and low power of our study.

DIC affects not only the anticoagulation system, but also the fibrinolytic system. Yet, data on the fibrinolytic approach are limited. R-TPA has been used in 3 infants with DIC due to meningococcemia [20,24] with improved perfusion and cardiac performance. Only one study [11], performed in an adult rabbit model of DIC, utilizing R-TPA and R-hirudin, addressed both anticoagulation and fibrinolytic abnormalities and reported improved hemostatic markers and decreased fibrin deposits. To our knowledge, our study represents the first attempt to utilize dual treatment of DIC in the susceptible neonate or animal model representative of the neonate. Our major goal was to determine if addressing both sides of the problem in the neonatal pig after endotoxin-induced DIC could improve survival and outcome.

## Conclusion

Our very preliminary data suggests that combined therapy with AT and R-TPA improved survival, degree of macroscopic and microscopic organ involvement and hematocrit in this piglet model of endotoxin-induced DIC compared with supportive care alone. We were unable to demonstrate such beneficial effects with the use of single agent therapies, but this may be due to the small sample size resulting in low power. AT administration increased AT levels similarly whether used as a single agent or in combination with R-TPA. Yet, the beneficial effect on survival and organ involvement was only significant in the combined therapy group suggesting an additive effect of the fibrinolytic component. No adverse effects, e.g. exacerbation of bleeding or clot formation, were found using these dosages in this model. Further studies with larger numbers of subjects are obviously needed to confirm these findings and to assess the true safety and efficacy of the combined therapeutic approach. However, the findings are encouraging and may point the way to a novel therapeutic approach to improve mortality and the quality of life for neonates that survive an episode of DIC.

## Competing interests

R-TPA was provided for experiment as a grant through Genentech, Inc.

## Authors' contributions

RDJ developed the concept for this research project, performed the experiments, collected and compiled data, performed certain specialty assays, took photographs and wrote the manuscript.

HC evaluated histopathological slides of postmortem tissues, developed the grading grading system and reported findings in a blinded fashion.

RH performed statistical analysis on the data and provided statistical expertise in interpreting the data.

HSK provided expertise in the gastrointestinal aspects of the study and inspected postmortem gastrointestinal tissues for ischemia and infarcts.

KM provided equipment and expertise in cardiovascular and blood pressure monitoring aspects of the experiment as well as interpretation of the data dealing with the above.

CD aided in the performance of the specialty assays and the performance of experiments.

RG provided expertise in hematological aspects of the study, aided in performing experiments and in the interpretation of data

DP provided expertise in neonatal medicine and pig physiology. She mentored the process of developing the concept into a bench project, performed experiments in conjunction with RDJ, took photographs and advised in the writing of the manuscript.
